# Integrated multimodal hierarchical fusion and meta-learning for enhanced molecular property prediction

**DOI:** 10.1093/bib/bbaf251

**Published:** 2025-05-30

**Authors:** Xianjun Han, Zhenglong Zhang, Can Bai, Zijian Wu

**Affiliations:** School of Computer Science and Technology, Anhui University, Jiulong Road 111, Hefei 230601, China; School of Computer Science and Technology, Anhui University, Jiulong Road 111, Hefei 230601, China; School of Acupuncture and Tuina, Anhui University of Chinese Medicine, Longzihu Road 350, Hefei 230012, China; School of Acupuncture and Tuina, Anhui University of Chinese Medicine, Longzihu Road 350, Hefei 230012, China

**Keywords:** molecular property prediction, multimodal fusion, meta-learning algorithm, molecular graph

## Abstract

Accurately predicting the pharmacological and toxicological properties of molecules is a critical step in the drug development process. Owing to the heterogeneity of molecular property prediction tasks, most of the current methods rely on building a base model and fine-tuning it to address specific properties. However, constructing a high-quality base model is a time-consuming procedure and requires a carefully designed network architecture; in addition, in certain rare molecular property prediction tasks, the base model often does not transfer well to new tasks. In this work, we adopt a meta-learning-based training framework that enables our model to adapt to diverse tasks with limited data, thereby preventing data scarcity from impacting certain molecular property predictions. Additionally, this framework leverages the correlations between different tasks, allowing the constructed model to quickly adapt to new prediction tasks. Moreover, we propose a multimodal fusion framework that combines two-dimensional molecular graphs with molecular images. In the molecular graphs, node-, motif-, and graph-level features are hierarchically guided from low to high levels, fully exploiting the molecular representation and more efficiently conducting hierarchical fusion. Experimental results indicate that our model outperforms the baseline models across various performance indicators, thereby validating the effectiveness of our approach.

## Introduction

In deep learning-based molecular property prediction tasks [1], accurately representing molecular structures is crucial for achieving high-precision predictions. Many studies employ natural language models [2] to process molecular string representations such as simplified molecular input line entry system (SMILES) [3, 4] and SELFIES representations [5]. However, these one-dimensional representation methods cannot precisely describe the topological structures of molecules [6]. Consequently, an increasing number of researchers are focusing on two-dimensional graph neural networks (GNNs) [7, 8], which can effectively learn the representations of nodes (i.e. atoms) and edges (i.e. chemical bonds) [9, 10] and update these atomic representations by aggregating information from neighboring nodes. Similarly, Masumshah *et al*. [11] explored neural network-based methods for molecular property prediction, further highlighting the effectiveness of machine learning models in learning molecular representations.

Although molecular graphs can provide the connectivity and local structural information between atoms, the complexity of some molecular structures far exceeds the description capabilities of simple molecular graphs [12]. Employing multimodal data fusion strategies for molecular property prediction can increase the resulting accuracy. MolPROP [13], SMICLR [14], and DPSP [15] were successfully used to train a powerful multimodal molecular model by combining SMILES and two molecular graph modalities. Compared with the fusion of molecular graphs and SMILES sequences in different dimensions, the integration of molecular graphs and molecular images in the same dimension has significant natural advantages. Ana *et al*. [16] effectively improved the ability of their model to understand molecular characteristics by pretraining on microscopy images and the chemical structures of molecules. Similarly, Wang *et al*. [17] integrated molecular graphs and molecular images to increase the accuracy of molecular attribute prediction.

However, these methods are confined to atomic-level feature extraction tasks, overlooking the intricate network of relationships between atoms and higher level molecular properties. This limitation results in the insufficient consideration of the fine interactions between atoms, the nature of chemical bonds, and even the functionality and stability of overall molecular structures. Additionally, when facing diverse property prediction tasks, the current mainstream approach involves constructing a base model and then fine-tuning it for each specific attribute [18, 19]. Such methods [20, 21] are only responsible for extracting representations of molecules, and retraining and fine-tuning are required when switching between different molecular prediction tasks [22, 23]. This leads to three issues. First, constructing a high-quality base model is a time-consuming process that requires a carefully designed network architecture [24]. Second, when handling certain rare molecular property prediction tasks, the base model often fails to effectively transfer to new tasks. Finally, each fine-tuning iteration requires substantial computational resources and a significant amount of time, failing to fully leverage the correlations between different molecular property prediction tasks.

In summary, the existing molecular property prediction models focus excessively on acquiring specific task-related detail features while neglecting to construct a more broadly applicable task, thereby limiting their adaptability and generalizability in molecular property prediction scenarios. Meta-learning involves naturally constructing a more broadly applicable task framework, enhancing the generalizability of the developed model. It not only fully leverages the correlations between different tasks, thus effectively addressing the common data scarcity and task diversity issues in molecular property prediction cases, but also quickly adapts to new tasks, hence better generalizing to unseen molecular property prediction tasks and improving the accuracy and efficiency of the output predictions.

Hence, we introduce meta-learning strategies [25] that focus specifically on enhancing the ability of a model to efficiently learn and adapt to new tasks with limited samples [26]. We design a series of molecular property prediction tasks using the Reptile meta-learning algorithm [27] and randomly sample task instances from large datasets containing various molecular properties to train the model. This process enables the model to dynamically update its parameters when addressing different molecular property prediction tasks, gradually mastering more universal and cross-task transferable molecular feature representations.

Moreover, to obtain better molecular representations, we utilize a multimodal fusion framework that combines molecular graphs with molecular images. The model learns atom–bond relationships and identifies key molecular features such as aromatic rings and functional groups from the molecular graph structure. GNNs capture the roles of the local substructures within a whole molecule. Molecular images, on the other hand, provide more intuitive structural information, such as topologies and functional group distributions [28], and image models can learn features such as symmetries and bond angles that are difficult for GNNs to capture. Existing methods, such as Chemception, have demonstrated the effectiveness of molecular image-based modeling, achieving performance comparable with that of traditional QSAR models, whereas ImageMol [29] improved its transfer and generalization capabilities through self-supervised pretraining. Considering the facts that molecular graphs are sensitive to local environments and that molecular images assist in learning global structures, both modalities complement each other in a fusion framework, enhancing the performance of the resulting model [16]. Therefore, we fuse GNNs with image models to address data scarcity issues and achieve improved generalizability.

In a molecular graph, features derived from the node, motif, and graph levels are hierarchically guided from low to high, moving from basic atomic properties to a more complex hierarchical structure. The resulting hierarchical molecular graph explores the local structural details within the target molecule and the connection patterns between atoms, providing the model with a microscopic perspective at a fine level. Molecular images, with their intuitively displayed spatial configurations, further enrich the ability of the model to understand the macroscopic morphology and stereochemical characteristics of the molecule.

This integration of dual-modality information provides insights into not only the microstructures of molecules but also their overall macroscopic outlines, ensuring that the model can fully integrate the advantages of both modalities. Experimental results prove that our model demonstrates excellent performance in a series of molecular property prediction tasks, surpassing the current mainstream methods. The main contributions of this study are as follows.

- Integrating molecular images and molecular graphs via a multimodal approach ensures the effective extraction of molecular features.
- Hierarchical molecular graphs enable a deeper understanding of interactions and properties, thus enhancing the accuracy of molecular property prediction.
- Meta-learning enhances the generalizability of the model across various molecular property prediction tasks, alleviating the amount of required training data and enabling knowledge sharing between different tasks.

## Multimodal hierarchical fusion framework for molecular property prediction

We develop a multimodal fusion framework that combines two-dimensional molecular graphs with molecular images for property prediction. Our molecular graph processing strategy progresses from atomic nodes through motifs to the graph level, distilling microscopic features. Moreover, we construct an encoder–decoder structure that extracts macroscopic features from molecular images. We then combine these two feature types using multimodal fusion techniques for molecular property prediction purposes. The overall network architecture is shown in Fig. 2.

### Molecular graphs

In the proposed framework, the features of molecular graphs are composed of hierarchical characteristics stemming from the atom, motif, and graph layers.

#### Node level

At the atomic level, we transform molecules into graphs via RDkit [30]. The representation of a molecule is $G=\{V,E\}$, where $V$ is the set of nodes (atoms) $\{v_{0},v_{1}...v_{n}\}$ and $E$ is the set of edges (bonds) $\{e_{1},e_{2},...,e_{m}\}$. We perform one-hot encoding on the nodes and edges based on the properties of the atoms and bonds, respectively [31]. Their specific types and encoding lengths used are shown in Table 1. Thus, by considering properties such as the atomic type, atomic degree (number of connected edges), and formal atomic charge of each atom, a total of 31 dimensions are covered. After encoding them using one-hot encoding, these different attributes are concatenated to form the initial encoding of the target atom $\{ V\in R^ {n \times 31}\}$, where $n$ denotes the number of nodes. We subsequently map the initial 31-dimensional encodings of atoms to a 256-dimensional latent space via a linear layer, aiming to transform the raw information of atoms into a feature space that is conducive to subsequent computations.

**Table 1:** Initial encodings of the nodes and edges in the 2D molecular graph construction process

	Descriptor	Length
Node (atom)	the atomic type	11
	the degree of an atom	6
	the formal charge of an atom	5
	the chiral tag of an atom	4
	the hybridization of an atom	4
	whether an atom is aromatic or not	1
Edge (bond)	the bond type	4
	whether the bond is in a ring or not	1
	whether the bond is conjugated or not	1

#### Motif level

**Figure 1:**
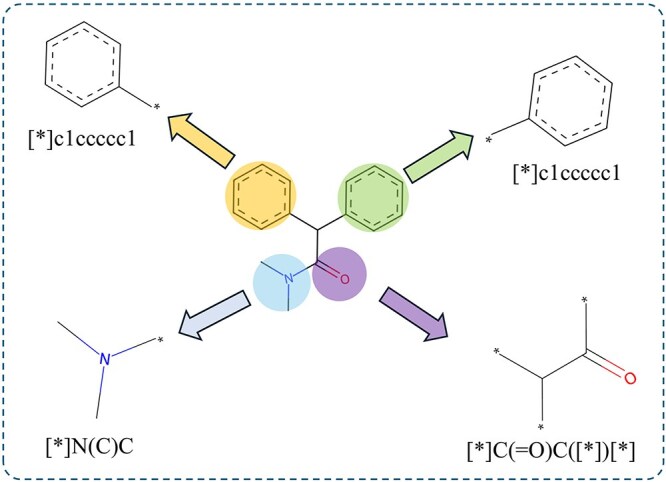
Illustration of the process of dividing molecular graphs into motifs.

Motifs, which are key structural units that repeatedly appear in molecules, contain important chemical information that determines molecular properties, which can enhance the ability of the model to understand and analyze complex chemical structures [22, 32]. We use the BRICS algorithm [33] to segment molecular structures. This algorithm identifies the potential breakpoints within a molecule, dividing it into multiple motif fragments, as shown in Fig. 1. Large molecular structures are efficiently processed, and the key local features are accurately captured. Motif fragments are represented as independent nodes with an all-ones tensor as their initial embedding vector. Motif nodes aggregate atomic characteristics and convey this information to the molecular level, enhancing the understanding of the overall properties.

#### Graph level

We introduce a super node to capture the global features of molecules. This super node is initialized as a zero tensor and aggregates feature information from the motif layer during the GNN propagation process. This dynamic aggregation procedure ensures that the model can gradually form a profound understanding of the global characteristics of molecules.

As shown in Table 1, the types of chemical bonds, e.g. rings and conjugated bonds, contribute to the edges forming an $ n \times n \times 6 $ matrix ($\{ E \in R^ {n \times n \times 6}\}$, where $n$ denotes the number of nodes). In addition, owing to the hierarchical composition of nodes, the corresponding adjacency matrix also exhibits a hierarchical structure. Similar to nodes, we map the initial six-dimensional encodings of bonds to a 64-dimensional latent space through linear layers, providing the model with richer and more effective input features.

**Figure 2:**
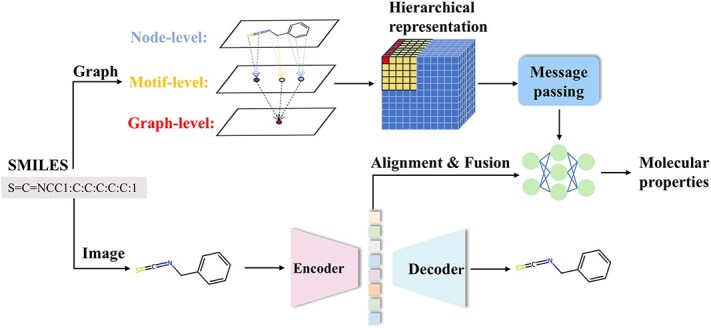
Overview of the multimodal hierarchical molecular property prediction framework.

A schematic diagram of the hierarchical molecular representation process is shown in Fig. 2. At the atomic level, the model focuses on depicting the basic physical and chemical properties of each atom and the fine interactions between neighboring atoms connected by chemical bonds. Moving up to the motif level, the emphasis shifts to analyzing the local structural units within the molecule that possess specific functions or reactivities. Upon reaching the graph level, the super node integration mechanism enables the integration of features across different hierarchies. This hierarchical architectural design captures and integrates multidimensional feature information at the atomic, motif, and molecular levels, endowing the model with powerful multiscale analysis capabilities.

#### Message passing

**Figure 3:**
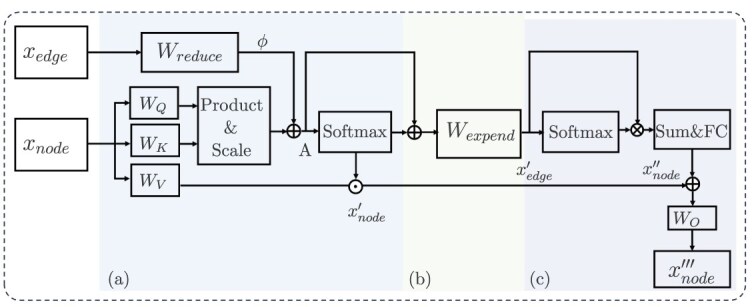
The graph propagation transformer aggregates information for message passing.

We employ a graph propagation transformer (GPTrans) [34] to aggregate information across the different hierarchical levels of molecules. Leveraging self-attention mechanisms, the GPTrans incorporates three propagation paths: node-to-node, node-to-edge, and edge-to-node pathways. Through message passing, the nodes at each layer can thoroughly learn the latent features of edges and their neighboring nodes, extracting a comprehensive representation of the molecule. The structure of the GPTrans is shown in Fig. 3.

As shown, we use parameter matrices $W_{K}$, $W_{Q}$, and $W_{v}$ to project the node embeddings $x_{\text{node}}$ to keys $K$, queries $Q$ and values $V$: 


(1) \begin{align*}& K=x_{\text{node}}W_{K}, Q=x_{\text{node}}W_{Q}, V=x_{\text{node}}W_{V}\end{align*}

The parameter matrix $W_{reduce}\in R^{d \times n_{head}}$ is subsequently used to predict layer-specific attention biases from the edge embeddings $x_{\text{edge}}$: 


(2) \begin{align*}& \phi =x_{\text{edge}}W_{\text{reduce}},\end{align*}

where $d=64,n_{head}=4$. Subsequently, we add $\phi $ to the attention map of the query-key dot product and compute the updated node embeddings $x_{\text{node}}^{\prime }$: 


(3) \begin{align*}& A=\frac{QK^{T}}{\sqrt{d_{head}}}+\phi, x_{\text{node}}^{\prime}=\text{softmax}(A)V.\end{align*}

Here, $d_{head}=4$, $x^\prime _{node}$ is the updated node embedding, and $\text{softmax}$ represents the activation function.

To incorporate node features into the edge features, we must also project the node features into a dimension consistent with that of the edge features: 


(4) \begin{align*}& x_{\text{edge}}^{\prime}=(A+\text{softmax}(A))W_{\text{expend}},\end{align*}

where $W_{expand}$ represents the parameter matrices and $x_{\text{edge}}^{\prime }$ represents the updated edge features obtained after the node features are fused. Similarly, we reincorporate the edge features back into the node features: 


(5) \begin{align*}& x_{\text{node}}^{\prime\prime}=\text{FC(sum}(x_{\text{edge}}^{\prime}\cdot\text{softmax}(x_{\text{edge}}^{\prime}),\text{dim=1})).\end{align*}

Here, $(\cdot )$ denotes the elementwise multiplication operation, and $\text{sum()}$ represents the process of summing the edge features along the second dimension, which can reduce the dimensionality of the edge features to match that of the node features. A fully connected (FC) layer is used to align the dimensions of the edge features with those of the node features. Finally, the node features obtained after each update are aggregated via residuals to yield the final features for each node: 


(6) \begin{align*}& x_{\text{node}}^{\prime\prime\prime}=\left(x_{\text{node}}^{\prime}+x_{\text{node}}^{\prime\prime}\right)W_{O},\end{align*}

where $W_{O}\in R^{256 \times 256}$ is a learnable matrix for implementing node feature mapping and $x_{\text{node}}^{\prime \prime \prime }$ denotes the final updated node features obtained after completing the aggregation step. In our experiments, we obtain features for all atoms through three iterations of updates. Additionally, to streamline the computations, we adopt the features derived from the graph level as the ultimate representation of the molecular graph, which is then used for multimodal integration purposes.

### Molecular images

RDKit-generated molecular images are 2D rendered images that are optimized by force fields to determine atom positions. Compared with molecular graphs, they provide additional information: (1) the geometric arrangement determines how atoms are arranged in ring or chain structures; (2) spatial conformation shows stereochemical information such as cis-trans isomerism and chiral centers; (3) molecular symmetry reflects the overall shape and symmetry features; and (4) global topological features represent overall relationships, whereas molecular graphs focus on local relationships.

In the proposed multimodal molecular property prediction framework, we integrate macroscopic features derived from molecular images and microscopic features acquired from hierarchical molecular graphs. Hence, an encoder–decoder architecture is tailored to extract features from molecular images. The overall architectural design of the encoder is illustrated in Table 2. The decoder structure is the reverse of the encoder structure.

**Table 2:** Network architecture of the molecular image encoder, where BN denotes batch normalization, ks denotes the kernel size, st denotes the stride, and pa denotes the padding

Layer name	Output size	Layer configs
Conv&BN&ReLU	64^*^224^*^224	ks=3, st=1, pa=1
AvgPool2d	64^*^112^*^112	ks=2, st=2, pa=0
Conv&BN&ReLU	128^*^112^*^112	ks=3, st=1, pa=1
AvgPool2d	128^*^56^*^56	ks=2, st=2, pa=0
Conv&BN&ReLU	256^*^56^*^56	ks=3, st=1, pa=1
AvgPool2d&BN&ReLU	256^*^28^*^28	ks=2, st=2, pa=0
Conv&BN&ReLU	1^*^28^*^28	ks=3, st=1, pa=1
Transformer&Linear	1^*^768	
Conv&BN&ReLU	768^*^28^*^28	ks=3, st=1, pa=1
Conv&BN&ReLU	1^*^28^*^28	ks=1, st=1, pa=1
Conv&BN&ReLU	2048^*^28^*^28	ks=1, st=1, pa=1
Conv&BN&ReLU	1^*^28^*^28	ks=1, st=1, pa=1
Linear1	1^*^784	bias=True
Linear2	1^*^768	bias=True
Conv&BN&ReLU	3^*^28^*^28	ks=3, st=1, pa=1
Upsampling	3^*^56^*^56	
Conv&BN&ReLU	3^*^56^*^56	ks=3, st=1, pa=1
Upsampling	3^*^112^*^112	
Conv&BN&ReLU	3^*^112^*^112	ks=1, st=1
Upsampling	3^*^224^*^224	

First, we utilize the RDKit tool [30] to transform SMILES strings into 2D molecular images and employ a reconstruction method to extract deep features from these images. During the encoding phase, convolutional kernels of various sizes are used to extract features from the molecular images, capturing characteristics across different scales. Subsequently, we employ a convolutional neural network (CNN)-style self-attention mechanism [35] to learn the interrelationships between different regions of the molecular images: 


(7) \begin{align*}& \begin{split} Q_{i,j}=\Sigma_{l=-1}^{1}\Sigma_{g=-1}^{1}E_{2+l,2+g}^{q}x_{i+l,j+g}\\ K_{i,j}=\Sigma_{l=-1}^{1}\Sigma_{g=-1}^{1}E_{2+l,2+g}^{k}x_{i+l,j+g}\\ V_{i,j}=\Sigma_{l=-1}^{1}\Sigma_{g=-1}^{1}E_{2+l,2+g}^{v}x_{i+l,j+g}. \end{split}\end{align*}

Here, (i,j) represents the pixel coordinates of the feature map $x$; $E^{q}$, $E^{k}$, and $E^{v}$ are all convolutional kernels; for any pixel point $(m, n)$, its similarity score $I_{m,n}^{i,j}$ with respect to $(i, j)$ can be calculated via the following expression: 


(8) \begin{align*}& I_{m,n}^{i,j}=\frac{\Sigma_{l=0}^{c_{q}}Q_{i,j}K_{m,n}}{\sqrt{\Sigma_{l=0}^{c_{q}}Q_{i,j}^{2}\sqrt{\textstyle\Sigma_{l=0}^{c_{q}}K_{m,n}^{2}}}},\end{align*}

where $c_{q}$ denotes the number of channels contained in feature map $Q$. Moreover, we introduce a learnable Gaussian distance matrix $M$: 


(9) \begin{align*}& M_{m,n}^{i,j}=e^{-\frac{(i-m)^{2}(2d_{/H})^{2}+(j-n)^{2}(2^{d}/W)^{2}}{2(\theta\times\alpha)^{2}}},\end{align*}

where $H$ and $W$ correspond to the height and width of the feature map, respectively; $d$ is the number of pooling layers, with each layer halving the size of the feature map; $\theta $ is a learnable parameter; and $\alpha $ is an adjustable hyperparameter.

Next, the similarity score feature map $I$ is elementwise multiplied with a Gaussian distance matrix $M$ to obtain a distance-reallocated feature map $A$. Feature map A incorporates the positional information of pixel points, meaning that higher weights are given to pixel points that are closer in terms of distance: 


(10) \begin{align*}& A_{i,j} = I_{i,j} \times M_{i,j}.\end{align*}

Next, the features are further processed through flattening and linear layers to obtain the deep latent features of the molecular images: 


(11) \begin{align*}& MolImg_{embeddings} = \text{FC}(\text{Flatten}(A\times V)),\end{align*}

where $Flatten$ refers to the flattening operation, which aims to map the $28 \times 28$ feature map to a $1 \times 784$-dimensional vector, and FC denotes an FC layer with dimensions of $ 768 \times 784$. Compared with a vision transformer [36], which adopts an additional positional encoding process, this module preserves positional information while enhancing the ability of the model to recognize and understand the structural configurations of the functional groups contained in molecules.

In the decoding stage, we restore the $1 \times 784$-dimensional embedding back to a $28 \times 28$-dimensional feature map and then progressively upsample it to recover a molecular image with a size of $224 \times 224$. We use the mean squared error as a loss function to optimize the model. Once the training process is complete, we fix the parameters of the encoder so that it is only used to extract the embedding vectors of the molecular images during subsequent processes. The features learned by the model from the molecular graphs and molecular images are summarized in Table 3.

**Table 3:** Comparison between the features learned from molecular graph structures and molecular images

Data source	Main learning features	Contribution to prediction tasks
Molecular graph	Atom type	Affects the physicochemical properties of molecules, such as their polarity and solubility
	Bond type	Determines conjugated systems, charge distributions, etc.
	Local topological structure	Key functional groups determine biological activity and reactivity
	Substructure	Plays different roles in various prediction tasks (e.g. toxicity and activity)
Molecular image	Symmetry	Influences the chiral activity and reaction selectivity of molecules
	Global topological structure	Affects the solubility and diffusivity of molecules
	Spatial distribution features	Impact electron density distributions and intermolecular interactions
	Conjugated system distribution	Influences light absorption and redox properties
	ring strain	Affects chemical reactivity and stability

### Alignment, fusion, and prediction

We choose the bottleneck attention module (BAM) [37] as the attention-enhanced fusion module to effectively integrate $x_{\text{super}}$ and $\text{latent}$ while reducing the redundancy between molecular graph features and image features. The BAM is a lightweight and efficient attention mechanism that was designed specifically for feedforward CNNs. By introducing attention mechanisms in both the channel and spatial dimensions, the BAM enhances the representation ability of the constructed model. This module is integrated into the intermediate layers of the feature downsampling mechanism, constructing hierarchical attention while maintaining a low parameter count. Additionally, the BAM can be seamlessly incorporated into any feedforward model, supporting end-to-end training and being widely applicable to various network architectures. Specifically, we map the molecular graph features extracted by the two modules to the same latent space: 


(12) \begin{align*}& x_{\text{graph}} = W_{g} x_{\text{super}}, \quad x_{\text{image}} = W_{i} x_{\text{latent}}.\end{align*}

Next, we concatenate the aligned features and input them into the BAM for attention enhancement purposes: 


(13) \begin{align*}& x_{\text{concat}} = \text{Concat}(x_{\text{graph}}, x_{\text{image}})\end{align*}

The BAM module leverages channel and spatial attention mechanisms to capture the key modality correlations and dynamically adjust the weights of different modalities, thereby extracting crucial information for molecular property prediction. The fused feature representation can be expressed as follows: 


(14) \begin{align*}& x_{\text{fused}} = \text{BAM}(x_{\text{concat}}).\end{align*}

Finally, we employ a multilayer perceptron layer to map the fused features to the final prediction output: 


(15) \begin{align*}& \hat{y} = \sigma(W_{o} x_{\text{fused}} + b_{o}).\end{align*}

Here, $W_{o} \in \mathbb{R}^{d^{\prime} \times C}$, $b_{o} \in \mathbb{R}^{C}$, and $\sigma (\cdot )$ is the activation function.

Through the mapping process of the FC layer in Equation 11, the model has already aligned the image and graph features. We concatenate the features extracted from the multilevel molecular graph (256 dimensions) with the molecular image embedding (768 dimensions), resulting in a composite molecular representation consisting of 1024 dimensions. We subsequently map this 1024-dimensional feature vector to a 64-dimensional vector through two linear layers, where the resulting vector is used for predicting various molecular properties. This enriches the understanding of molecular characteristics, deepens the understanding of properties, and provides a comprehensive perspective for accurately performing prediction. During training, we use the binary cross-entropy loss to quantify the discrepancies between the predicted and actual properties.

## Optimizing the network with the Reptile meta-learning algorithm

We employ a meta-learning-based training framework that bolsters the efficiency of the model across various molecular property prediction scenarios, incorporating a broad spectrum of diverse tasks during the model training phase. Consequently, this step empowers the model with dynamic capabilities, allowing it to rapidly adapt and respond to emerging prediction tasks, thereby ensuring the generalizability and flexibility of the model.

We utilize the Reptile meta-learning algorithm [27] to optimize our model. Reptile is a first-order gradient-based meta-learning algorithm. Its core concept involves updating the initial model parameters by performing gradient descent across multiple tasks. Initially, the model parameters are randomly initialized as meta-learners $\theta $. Within the constructed multitask dataset $\rho (T)$, we sample several similar molecular property prediction tasks $t$ (2 ways, 3 shots). The meta-learning framework typically divides tasks into a support set and a query set to simulate the training and testing processes of the model in a few-shot setting. The support set provides a small amount of labeled data, enabling the model to quickly adjust its parameters, whereas the query set is used to evaluate the generalizability of the model after it sees only a few samples. This division scheme originated from classic few-shot learning tasks such as Kinomemeta [38] and Meta-MolNet [39] and has been widely validated.

**Figure 4:**
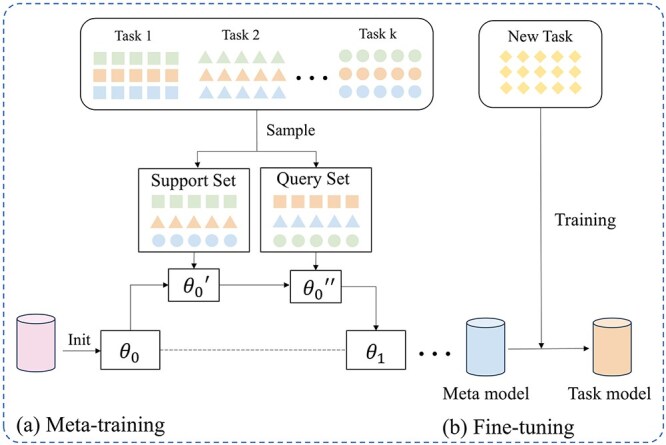
Illustration of the network optimization process implemented via the Reptile meta-learning algorithm.

Concurrently, the support set ($S_{t}$, $Y_{t}$) and query set ($Q_{t}$, $Y_{t}^{\prime }$) are randomly partitioned at a $1:5$ ratio. During each update iteration, the meta-model is updated on the basis of the support set $\{G_{S_{t_{1}}},G_{S_{t_{2}}},...G_{S_{t_{k}}}\}$ of each task, performing three gradient descent steps with a learning rate of $\alpha $. This results in base learners $Model$ that are specific to each task, with parameters $\theta ^{\prime }$. The base learners then calculate the losses induced on the query sets $\{G_{Q_{t_{1}}},G_{Q_{t_{2}}},...G_{Q_{t_{n}}}\}$ of their respective tasks and update the models with a learning rate of $\beta $, yielding the final model $Model^{\prime }$ with parameters $\theta ^{\prime \prime }$. Finally, the direction of the parameter difference $\theta ^{\prime \prime }-\theta $ between the final model $Model^{\prime }$ and the meta-model $Model$ is used for updating the meta-model. The process of optimizing the network using the Reptile meta-learning algorithm is shown in Fig. 4, and the specific algorithm is as follows:


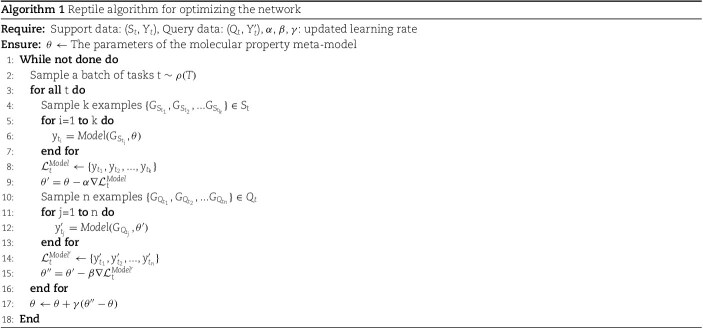


Notably, when significant differences are present between tasks, the generalization ability of the meta-model may be limited, which is a common challenge faced by meta-learning methods. To adapt to these tasks, the following conditions must be satisfied. First, task type consistency is needed; the current model is trained and evaluated only on binary classification tasks, and if it is applied to regression tasks, the model structure and training mechanism need to be adjusted accordingly. Second, task semantic relevance is necessary; the five tasks selected herein are all related to drug properties (toxicity, solubility, inhibition activity, etc.). Although these properties differ, they share common underlying drivers related to molecular structures. If the semantic difference between two tasks is large (e.g. switching from toxicity prediction to reaction yield prediction), the model may struggle to extract task-agnostic representations, leading to decreased generalizability. Finally, data distribution similarity is essential; the datasets used in this work consist of small-molecule drug compounds, which have similar atomic compositions, molecular scales, and graph structure distributions. If the current model is applied to molecules with significantly different structures, such as natural products, metal–organic frameworks, or large molecules including proteins, it may not transfer effectively.

## Experimental results

### Data processing and parameter settings

**Figure 5:**
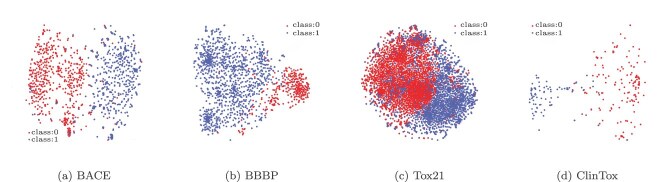
Molecular representation visualization diagrams, where each point represents a molecular representation in two-dimensional space.

We utilized MoleculeNet benchmark datasets [40], which cover key pharmaceutical properties such as BACE (evaluating binding affinities for $\beta $-secretase Bace1 inhibitors), Tox21 (predicting 21 different toxicity endpoints) [41], HIV (assessing the bioactivity of HIV inhibitors), BBBP (predicting blood–brain barrier permeability), and ClinTox (examining the toxic characteristics of clinical drugs) [42], to train our models (Fig. 5). These diverse datasets ensured that our models could comprehensively understand and accurately predict multiple properties of drug molecules.

We conducted a detailed statistical analysis of the training data and meticulously partitioned the dataset into training, validation, and test sets via the scaffold splitting method, with proportions of $80\%$, $10\%$, and $10\%$, respectively.

Next, we combined the data for these diverse tasks into a multitask dataset by removing duplicate samples to ensure the uniqueness of each sample contained in the dataset. Through these preprocessing steps, we successfully constructed a reliable and informative multitask dataset, enhancing the ability of the model to generalize.

In the experiment, we used an NVIDIA GeForce GTX 1650 GPU to train the model. The meta-learning-based training parameters were set as follows: a total of 1000 batches with a fixed learning rate of 0.001 were used, each sampling step included three tasks for joint training, the support set underwent 5 iterations, and the entire process was cycled 10 times. The final model output was selected on the basis of the optimal training performance to ensure peak performance.

### Evaluation metrics and results comparison

We employed the area under the receiver operating characteristic curve (ROC-AUC) as a metric to evaluate the classification performance of the proposed model. The ROC-AUC is a common evaluation index that is used to measure the capabilities of a binary classifier across all possible thresholds. By plotting the relationship between the true-positive rate and false-positive rate, we can visually assess the classification accuracy of the model. The higher the ROC-AUC value is, the better the classification performance of the model.

To evaluate the effectiveness of our proposed model, we compared it with several leading molecular property prediction methods with excellent performance. These methods are described as follows. GIN [7] captures complex relationships and node features in graph structures through deep learning, making it highly adaptable to new tasks. AttMask [43] applies random masking and self-supervised learning to predict missing attributes. GPTGNN [44] uses self-supervised learning and random masking techniques to enhance its graph representation and generalization capabilities. ConPred [43] generates context node pairs to learn global relationships. InfoG [45] maximizes the interaction information between node pairs to capture local and global structures. MoCL [21] combines multiple self-supervised tasks to learn multilevel representations. GrLoG [46] enhances its learning capabilities through logical graph reasoning tasks. GraphCL [47] improves the resulting representation quality through contrastive learning. JOAO [48] uses self-supervised learning objectives to enhance its molecular property prediction accuracy. MolCLR [49] maximizes the similarity between similar molecular graph pairs through contrastive learning. G_Motif [50] learns substructure patterns to capture local graph structures. MGSSL [32] optimizes multilevel representations by combining multiple self-supervised tasks. G_SAGE [8] samples neighbor nodes to improve its computational efficiency and handle large-scale graph datasets. MetaGIN [51] employs a lightweight meta-learning-based GNN framework to enhance its molecular property prediction performance in small-sample scenarios. It leverages the Reptile algorithm to conduct cross-task model parameter initialization, enabling it to rapidly adapt to new molecular tasks. MMGCF [52] introduces a counterfactual explanation generation framework for molecular property prediction, with an emphasis on model interpretability. This approach reconstructs substructures (motifs) in molecular graphs, generating molecules with similar structures but different predicted outcomes by replacing key substructures. Himol [22] uses a multilevel molecular graph structure and random masking strategies to improve its attribute prediction accuracy. These methods have advanced the field of molecular property prediction and have achieved excellent predictive performance.

By utilizing a shared dataset for training, we evaluated the performance of the proposed method in comparison with that of competing methods on the basis of their best-reported outcomes. To assess the robust generalization ability of our meta-learning model, we directly applied the pretrained model without fine-tuning it on specific datasets. As shown in the penultimate row of Table 4, the baseline models (such as GIN, GraphCL, and MolCLR) were trained independently on each dataset. The ”Meta-model” shown in the table refers to our proposed meta-learning model, which was trained simultaneously across five datasets. It learned task-agnostic representations and initial parameter through a shared meta-learner. ”Ours” in the table refers to the final model obtained by fine-tuning the meta-initialized weights acquired from the ”Meta-model” on each dataset with few adjustments. The meta-model was able to achieve similar or better performance than that of some baseline models without the need to train a separate model for each task, demonstrating its excellent cross-task generalizability and fully proving the superiority of the meta-learner. Our model, which was further fine-tuned based on the meta-model, significantly outperformed the baseline methods. This indicates that the meta-model provided a better initial state, allowing it to achieve outstanding performance in each task with only a few training steps.

**Table 4:** Comparison among the ROC-AUC values attained by different methods in molecular property prediction tasks

Model	#Param	BACE	BBBP	HIV	ClinTox	Tox21	Avg.
GIN [7]	5.1M	0.72	0.70	0.69	0.61	0.75	0.69
AttMask [43]	5.2M	0.79	0.64	0.65	0.61	0.74	0.68
GPTGNN [44]	5.5M	0.72	0.69	0.72	0.69	0.73	0.69
ConPred [43]	8.5M	0.77	0.68	0.69	0.71	0.72	0.71
InfoG [45]	6.5M	0.76	0.69	0.73	0.73	0.75	0.73
MoCL [21]	10.5M	0.75	0.66	0.69	0.60	0.71	0.68
GrLoG [46]	11M	0.79	0.65	0.61	0.73	0.73	0.70
GraphCL [47]	7.5M	0.73	0.67	0.71	0.78	0.75	0.73
JOAO [48]	13M	0.72	0.70	0.66	0.79	0.75	0.72
MolCLR [49]	9.2M	0.76	0.69	0.71	**0.83**	0.74	0.75
G_Motif [50]	7.3M	**0.83**	0.68	0.72	0.78	0.73	0.75
MGSSL [32]	15M	0.79	0.69	0.74	0.80	0.76	0.76
MetaGIN [51]	17M	-	0.80	-	**0.83**	0.78	-
MMGCF [52]	-	-	**0.85**	0.80	-	-	-
G_SAGE [8]	5.0M	0.72	0.67	0.68	0.52	0.69	0.66
Himol(S) [22]	3.4M	0.78	0.68	0.72	0.70	0.71	0.72
Himol(L) [22]	5.5M	0.79	0.71	0.78	0.80	0.74	0.76
Meta-model	**0.3M**	0.78	0.74	0.74	0.75	0.76	0.75
Ours	**0.3M**	**0.83**	0.78	**0.82**	**0.83**	**0.79**	**0.81**

In addition, the meta-model without task-specific fine-tuning notably outperformed all the compared methods, which demonstrates the effectiveness of meta-learning in terms of enhancing the generalizability of models across different tasks. After a brief fine-tuning period of 10–20 epochs on the target dataset, our model achieved the best performance, as measured by the ROC-AUC metric.

Our approach employs a multilevel molecular graph framework that captures structural features at the atom, motif, and molecule levels. Unlike Himol, which overlooks the rich representational information contained in molecular images, our model incorporates a dedicated molecular image feature extraction module. This multimodal combination enhances molecular representations and provides more accurate information for predicting molecular properties. Additionally, our model conferred a significant advantage in terms of its parameter count. By employing a pretraining strategy and freezing the parameters of the image feature extraction module, we could significantly reduce the number of trainable parameters. The weight-sharing linear layers contained in the graph feature extraction module further decreased the number of required parameters. Ultimately, as shown in Table 4, the number of actual trainable parameters was only 0.3 M, representing a 91$\%$ reduction relative to the Himol(small) model, thus significantly reducing the imposed computational resource requirements and improving the training efficiency of our model.

### Impacts of the support and query set sizes

During meta-learning-based training, the sizes of the support and query sets are crucial for achieving good model performance. The support set is used to update the task model; if it is too small, the model may not adequately learn the characteristics of the task model, leading to insufficient generalization. Conversely, if it is too large, the model may overfit the input data, impacting its performance in new tasks and potentially preventing the meta-model from converging. The query set is used to evaluate the generalization ability of the model; ideally, it should be sufficiently large to accurately assess the performance of the model on new samples. However, an overly large query set may cause overfitting, thereby affecting the generalizability of the model. The division of these two sets directly impacts the training effectiveness of the model. During training, we optimized our model by adjusting its support and query set sizes, as detailed in Table 5, where the best results are indicated in bold font.

**Table 5:** The impacts of different support and query set sizes

Support set	Query set	Average ROC-AUC value
12	36	0.731
12	48	0.789
**12**	**60**	**0.808**
24	24	0.757
30	18	0.748
36	12	0.724
48	12	0.718

### Molecular representation visualization

To visually demonstrate the classification capabilities of the proposed model, we conducted a visualization experiment. Utilizing t-distributed stochastic neighbor embedding (t-SNE) nonlinear dimensionality reduction techniques [53], we mapped the 64-dimensional molecular representations extracted by our model to a two-dimensional space. In this technique, similar high-dimensional vectors are modeled as nearby points, whereas dissimilar vectors are modeled as distant points. From the visualization results shown in the figures, clear boundaries exist between the molecules with different labels from different datasets. Molecules with the same label tended to cluster together, whereas those with different labels are positioned farther apart. These findings indicate that the effective molecular representations extracted by our model can be used to accurately distinguish between different types of molecules.

### Meta-learning-based training performance analysis

**Figure 6:**
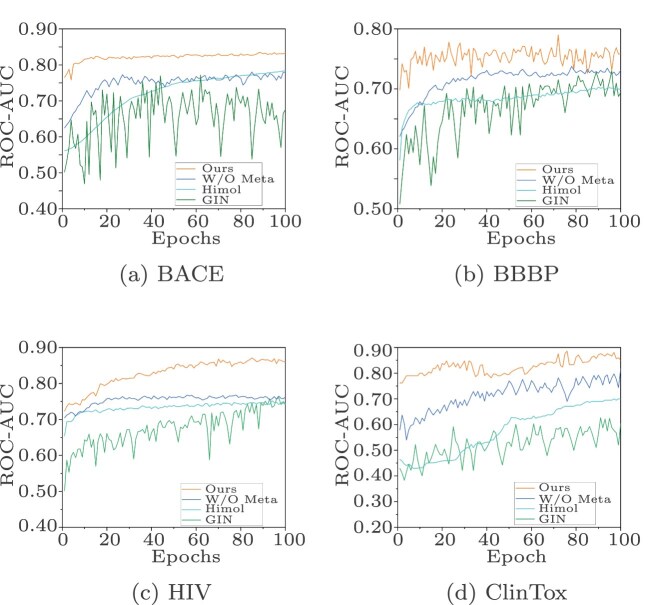
Classification ROC-AUC curves under different fine-tuning settings.

To validate the performance of our meta-learning model, we randomly selected four different datasets and compared the model with two leading molecular property prediction methods under the same experimental conditions, where the ROC-AUC metric was used to measure the classification accuracy achieved at each training epoch. As shown in Fig. 6, GIN [7] had a slower convergence rate and required more training iterations to achieve good results. Additionally, during the training process, GIN exhibited poor stability and significant performance fluctuations. Despite attempting to layer molecular graphs, Himol [22] yielded limited precision improvements across the four datasets. In contrast, our method, even without meta-learning, outperformed GIN and Himol because it used multimodal fusion and hierarchical atomic feature extraction. After incorporating meta-learning, the classification accuracy of our model improved further because the meta-model could learn to quickly adapt to the patterns and features of different tasks and rapidly adjust its parameters on the basis of prior experience, thus significantly enhancing its generalization ability. Additionally, as shown in the figure, compared with the baseline model, our model converged faster, with the training time averaging only 40$\%$ of that of the baseline model. This fully demonstrates that our model significantly improves its training efficiency and effectively reduces its computational resource consumption level.

### Molecular image encoder visualization

**Figure 7:**
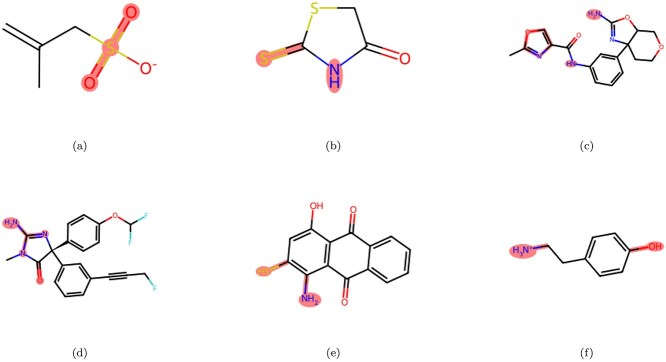
Feature visualization for the molecular image encoder.

To verify the feature extraction capabilities of the molecular image encoder, we normalized the extracted molecular feature maps and overlaid them with the original molecular images. This overlay operation enabled a visual inspection of whether the activation regions contained in the feature maps corresponded to key structures in the molecular images. As shown in Fig. 7, the image encoder focused primarily on the key chemical bonds or functional groups in the molecular images, effectively capturing the key visual features.

### Ablation study

**Figure 8:**
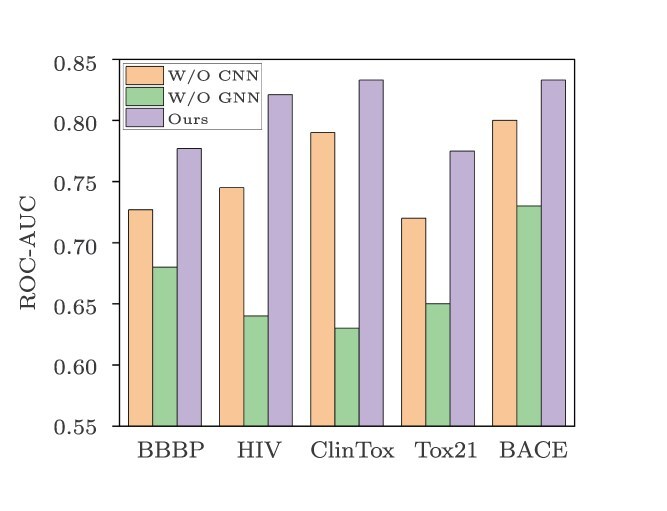
Results of ablation experiments, where the numbers represent ROC-AUC values.

To confirm the effectiveness of the molecular graph feature extraction module and the molecular image feature extraction module, we designed two ablation variants, i.e. W/O GNN and W/O CNN, as shown in Fig. 8.

In the W/O GNN experiment, we removed the molecular graph feature extraction module and used only the molecular image feature extraction module for feature extraction purposes. The results reveal a significant decrease in the predictive ability, highlighting the critical role of the molecular graph feature extraction module in capturing the deep relationships and interactions within molecular structures. This module converts atomic information into graph structures and learns the complex relationships between atoms via GNNs, enabling improved representations of molecular structure information to be obtained. In the W/O CNN experiment, we removed the molecular image feature extraction module and used only the molecular graph feature extraction module for feature extraction purposes. The results indicate that the molecular image feature extraction module is equally important for capturing molecular information. This module uses CNNs to learn local features from molecular images, converting two-dimensional molecular images into feature vectors and leading to improved molecular representations.

A comparison between the results of these two experiments reveals that the use of a single-modality feature extraction module limits the predictive ability of the model. Our multimodal model, which combines a molecular graph feature extraction module and a molecular image feature extraction module, more comprehensively captures molecular information, exhibits significantly improved predictive capabilities.

### COVID-19 dataset activity prediction

We further validated the proposed method on a COVID-19 activity prediction dataset. First, we used our model to predict the molecules in the dataset and evaluated its ability to distinguish between active and inactive compounds. Then, we applied t-SNE for performing dimensionality reduction and visualizing the obtained molecular representations to observe the ability of the model to distinguish between different molecular categories.

**Figure 9:**
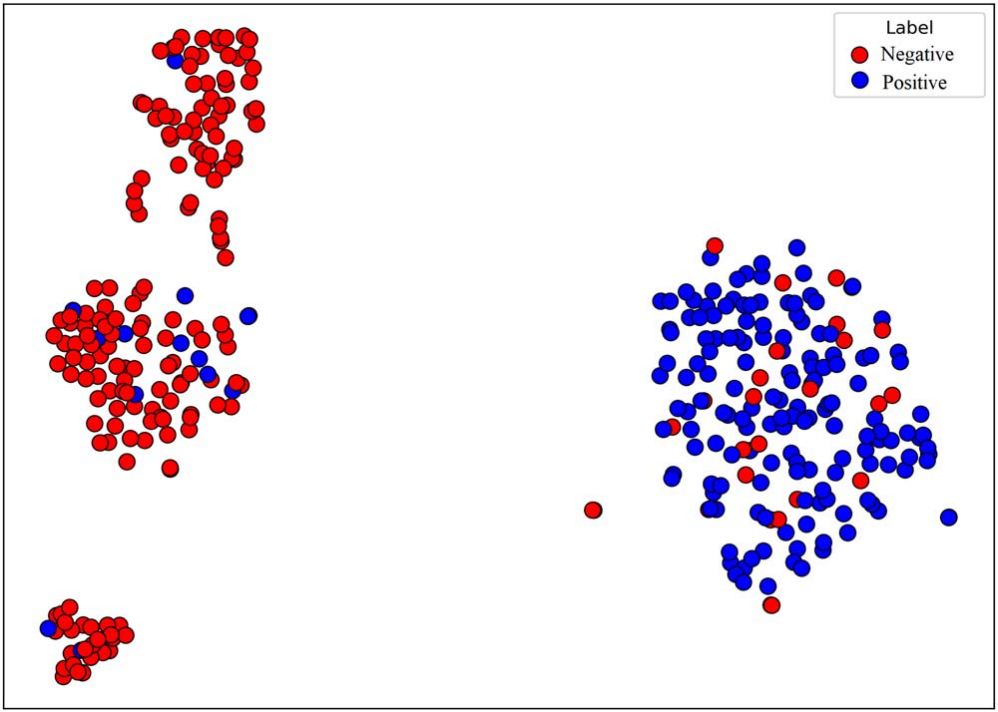
t-SNE visualization derived from the COVID-19 dataset.

As shown in Fig. 9, active compounds (positive class) and inactive compounds (negative class) formed distinct cluster structures in the two-dimensional projection space, indicating that the model effectively learned the molecular representations and accurately differentiated between different molecular categories. This result not only confirms the effectiveness of our method in terms of modeling complex viral targets and drug structures but also highlights its potential applicability for screening candidate drug molecules.

## Conclusion

A multimodal molecular property prediction framework that combines two-dimensional molecular graphs with molecular images is introduced in this paper. In the molecular graph domain, we construct a hierarchical molecular graph structure ranging from the node and motif levels to the graph level, providing a fine-grained microscopic perspective. Simultaneously, we employ a combination of CNNs and transformers to extract features from molecular images, capturing spatial structures from a macroscopic viewpoint. The integration of these dual modalities ensures that the model fully leverages the strengths of both modalities. During training, we utilize a meta-learning approach to incorporate diverse molecular property prediction tasks into a unified framework, significantly enhancing the generalizability and predictive accuracy of the model in molecular property prediction tasks.

## Future work

This work can be expanded in several areas in the future: (1) integrating 3D molecular structure information to better model spatial conformations, such as by incorporating 3D atomic coordinates or using conformational sampling methods; (2) exploring more advanced meta-learning strategies, such as MAML++, meta-stochastic gradient descent, or context encoder-based methods, to enhance the adaptability and generalizability of the model to low-resource tasks; (3) evaluating the cross-task generalization and transfer performance of the model, especially in more broadly distributed tasks such as pharmacokinetics and ADMET prediction; and (4) extending the model to real-world multitask joint learning scenarios for applications involving drug discovery.

**Key Points**

- A multimodal approach is adopted in this paper by combining molecular images and graphs, thereby facilitating the efficient extraction of molecular features.
- Hierarchical molecular graphs are leveraged in this paper to gain more profound insights into molecular interactions and properties, which in turn improves the precision of the resulting property predictions.
- Meta-learning techniques are applied in this paper to increase the generalizability of models across various molecular property prediction tasks, reducing the dependency on large training datasets and promoting cross-task knowledge sharing.

## Data Availability

The data and code are available at https://github.com/shiqi-senventeen/IMHF.
